# Evidence for Infection and Inflammation in Infant Deaths in a Country with Historically Low Incidences of Sudden Infant Death Syndrome

**DOI:** 10.3389/fimmu.2015.00389

**Published:** 2015-08-24

**Authors:** Klára Törő, Krisztina Vörös, Zsófia Mészner, Aletta Váradi-T, Adrienn Tóth, Katalin Kovács

**Affiliations:** ^1^Department of Forensic and Insurance Medicine, Semmelweis University, Budapest, Hungary; ^2^Department of Medical Chemistry, Molecular Biology and Pathobiochemistry, Semmelweis University, Budapest, Hungary; ^3^St. László Hospital for Infectious Diseases, National Institute of Child Health, Budapest, Hungary; ^4^Department of Pathology, Military Hospital – National Health Center, Budapest, Hungary; ^5^Hungarian Demographic Research Institute, Budapest, Hungary

**Keywords:** sudden infant death syndrome, infant mortality, immunization, infection, inflammation

## Abstract

Total infant mortality in Hungary has been higher than other European countries; however, the reported incidence of sudden infant death syndrome (SIDS) has been lower. The low incidence of SIDS in Hungary has been supported by evidence obtained from the high rate of scene of death investigation and medico-legal autopsy mandatory since the 1950s. In this study, we compared the incidence of explained and unexplained infant deaths in Hungary for three periods: 1979–1989 when the incidence of SIDS was high in western Europe; 1990–1999 when the incidence of infant deaths was falling following introduction of the public health campaigns to reduce the risk factors associated with SIDS; and 2000–2012 to determine if introduction of *Haemophilus influenzae* type b or pneumococcal vaccines or introduction of an earlier immunization schedule during this period had an effect on SIDS. Explained infant deaths fell consistently during this period; however, SIDS rose during the second period when the incidence of SIDS was falling in other European countries. Evidence for infection and/or inflammation was observed for the majority of SIDS during each period. The results are discussed in relation to campaigns to reduce infant mortality in Hungary and the introduction of new vaccines and an earlier immunization schedule in 2006.

## Introduction

**Figure 1:**
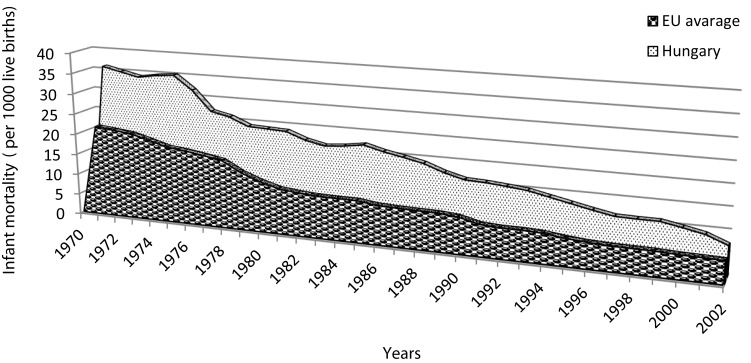
**Comparison of infant mortality (per 1000 live births) for Hungary and the European Union (data from the WHO Regional Office for Europe)**.

In 2004, we reported that infant mortality in Hungary was higher than many European countries (1) (Figure 1) and it has remained high according to the OECD figures for 2014 (2); however, the reported incidence of sudden infant death syndrome (SIDS) has been lower (0.15–0.3/1000 live births) than those of other countries (1, 3, 4). The historically low incidence of SIDS in Hungary has been supported by evidence obtained from the high rate of scene of death investigation and medico-legal autopsy.

It was reported that prior to 1980, the incidence of SIDS was lower in East Germany and this was attributed to a lower prevalence of prone sleeping compared with figures for West Germany (5). SIDS has many risk factors that parallel susceptibility to infectious diseases, and there is increasing evidence that infection and inflammatory responses play a role in the events leading to these deaths (Blackwell et al., this issue). During 1983–1989, the incidence of SIDS in Norway and Sweden appeared to parallel the incidence of whooping cough. Mortality rates followed significantly the monthly prevalence of *Bordetella pertussis* in Sweden where immunization for pertussis had been discontinued. In Norway where pertussis immunization was continued, this correlation was less obvious (6).

In relation to these observations, it has been suggested that the historic emphasis on infant immunization in Hungary might have contributed to the lower incidence of sudden deaths triggered by infectious agents. Immunization began in the late eighteenth century with variolization. Immunization with vaccinia replaced this practice, and the first mandatory vaccine for children in Hungary was for small pox in 1876. Tetanus toxoid was offered from 1940, but this was not mandatory.

A national immunization plan (NIP) has been in effect in Hungary since the mid-1950s and mandatory since its introduction; costs are covered by the national budget and exemptions are allowed only for medical reasons. The NIP began with universal BCG vaccine for infants at the maternity hospitals prior to discharge. This was followed by diphtheria-pertussis (whole cell)-tetanus (DPT), subsequently replaced in 2006 by an acellular pertussis vaccine. Immunizations were at 3, 4, and 5 months, with boosters at 3 and 6 years until 2006. Since then, the primary doses are given at 2, 3, and 4 months, and the boosters are given at 18 months and 6 years.

*Haemophilus influenzae* type b (Hib) prophylaxis began in 1999 (3, 4, and 5 months) in conjunction with DPT immunization. In 2006, the combined acellular DPT, inactivated polio and Hib vaccine was introduced. Pneumococcal conjugate vaccine (7-valent) was introduced in 2008 at 2, 4, and 18 months. It was free but not mandatory; however, the uptake was 83% for all eligible infants. The 7-valent vaccine was replaced by the 13-valent *n* in 2010.

The current study compared explained deaths due to infection and those due to SIDS in three periods: 1979–1989 when the incidence of SIDS was higher in western Europe; 1990–1999 when the incidence of infant deaths was falling following introduction of the public health campaigns to reduce the risk factors identified for SIDS (e.g., prevention of prone sleeping, overheating, and exposure to cigarette smoke); and 2000–2012 to determine if introduction of Hib or pneumococcal vaccines had an effect on SIDS. Available autopsy records were assessed for evidence of infection and inflammation among infant deaths in these three periods. As initiation of infant immunization in the United Kingdom was associated with a shift in the age ranges of SIDS infants (7, 8), data on age of SIDS infants in central Hungary were analyzed to determine if there was a similar decrease in the proportion of SIDS infants in the age range of 3–4 months following the change to the earlier immunization schedule.

## Materials and Methods

In Hungary since the 1950s, all sudden infant deaths are referred for medico-legal investigations and autopsy. Data on SIDS and deaths due to infection were collected from the Hungarian National Statistics Office Database for three periods: period 1, 1979–1989; period 2, 1990–1999; and period 3, 2000–2012. The ninth and tenth revisions of the international classification of diseases (ICD) were used for the determination of cause of death (Table 1). SIDS cases were classified as 798.0 (ICD version ninth) or R95 (ICD version tenth). ICD codes are derived from the autopsy records and death certificates. The ninth version covered the years between 1979 and 1995. The tenth version was introduced in 1996.

**Table 1:** **Selected ICD codes versions 9 and 10 reflected to the infections in different organs**.

ICD code version		
9	10	Cause of death
001–139	A00–B99	Certain infections and parasitic diseases
320–357	G00–G08	Diseases of the nervous system
360–389	H65–H70	Diseases of the ear and mastoid process
460–519	J01–J69	Diseases of the respiratory system
520–579	K35–K75	Diseases of the digestive system
580–629	N10–N39	Diseases of the genitourinary system
682–709	L00–L99	Diseases of the skin and subcutaneous tissue
710–739	M00–M99	Diseases of the musculoskeletal system
760–779	P23–P77	Certain conditions originating in the perinatal period
798.0	R95	Sudden infant death syndrome

Sudden infant death syndrome cases were diagnosed by the currently accepted definition (9). If severe inflammation or signs of septicemia were noted, these cases were not classified as SIDS. Infection and inflammation in different organ systems were analyzed and assigned to ICD codes (Table 1).

## Results

In the three time periods, there were 47412 infant deaths: period 1, 26124; period 2, 13433; and period 3, 7855. In each period, the majority of deaths were explained and a small number were classified as SIDS: period 1, 251 (0.9%); period 2, 331 (2.4%); and period 3, 271 (3.4%). For each period, there was an excess of male deaths among the SIDS infants: period 1, 155 (62%) male, 96 (38%) female); period 2, 193 (58%) male, 138 (42%) female; and period 3, 154 (57%) male, 117 (43%) female (Table 2). Most SIDS cases (70%) occurred between 2 and 6 months of age. The rate of mild infections among SIDS victim under 6 months old was 70%, while the proportion was only slightly lower (60%) among older SIDS infants.

**Table 2:** **Number of deaths during the three periods assessed by ICD classification and gender**.

Number of deaths (%)							
Selected ICD codes	1979–1989		1990–1999		2000–2012		1979–2012
	Male	Female	Male	Female	Male	Female	Male *+* Female
A00–B99	135 (0.9)	109 (1.0)	112 (1.5)	67 (1.2)	40 (0.9)	27 (0.8)	490 (1.0)
G00–G08	176 (1.2)	153 (1.4)	100 (1.3)	76 (1.3)	32 (0.7)	29 (0.8)	566 (1.2)
H65–H70	184 (1.2)	130 (1.2)	39 (0.5)	37 (0.6)	12 (0.3)	15 (0.4)	417 (0.9)
J01–J69	1144 (7.7)	797 (7.1)	423 (5.6)	262 (4.5)	111 (2.6)	82 (2.3)	2819 (5.9)
K35–K75	81 (0.5)	57 (0.5)	23 (0.3)	16 (0.3)	6 (0.1)	4 (0.1)	187 (0.4)
N10–N39	10 (0.1)	9 (0.1)	11 (0.1)	5 (0.1)	1 (0.0)	1 (0.0)	37 (0.1)
L00–L99	6 (0.0^a^)	4 (0.0^a^)	0 (0.0)	1 (0.0^a^)	0 (0.0)	0 (0.0)	11 (0.0^a^)
M00–M99	0 (0.0)	1 (0.0^a^)	0 (0.0)	0 (0.0)	1 (0.0^a^)	0 (0.0)	2 (0.0^a^)
P23–P77	488 (3.3)	361 (3.2)	235 (3.1)	184 (3.2)	235 (5.4)	205 (5.8)	1708 (3.6)
R95	155 (1.0)	96 (0.9)	193 (2.5)	138 (2.4)	154 (3.6)	117 (3.3)	853 (1.8)
TOT	14949	11175	7617	5816	4316	3539	47412

*^a^Percentage <0.1%*.

Respiratory infections (J01–J69) were the most common cause of death in all three periods; however, there was a steady decline, particularly after the introduction of the Hib vaccine in 1999 and the pneumococcal vaccine in 2006 (Figure 1). For each of the three periods, there was a higher proportion of males than females; however, the difference was not significant.

**Figure 2:**
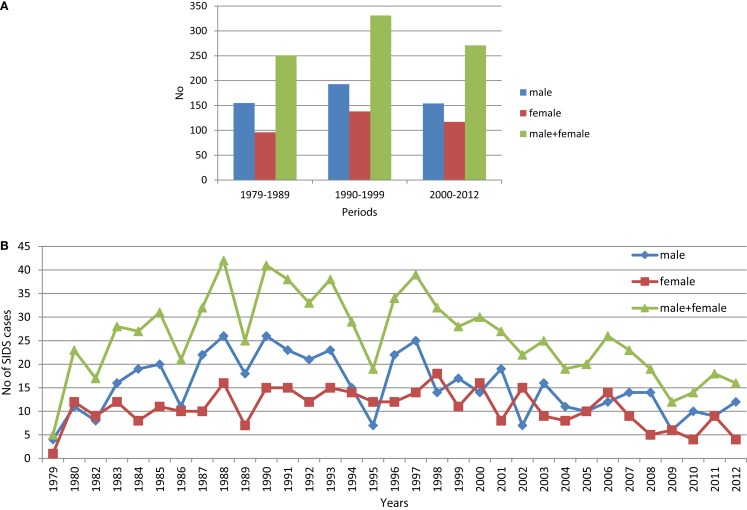
**Percentage distributions deaths due to respiratory diseases (J01–J69)**. **(A)** Number of SIDS cases in the three periods investigated. **(B)** Number of SIDS cases per years between 1979 and 2012.

**Figure 3:**
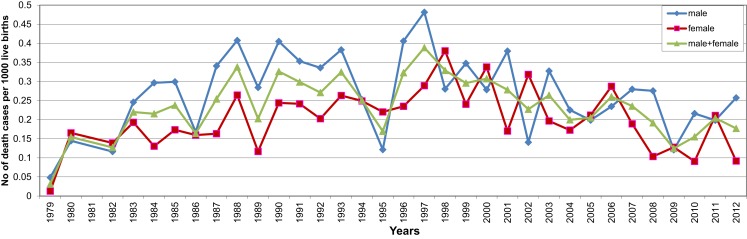
**Distribution of SIDS cases per 1000 live births 1979–2012**.

In contrast to the steady decline in deaths due to respiratory infections, there was an increase in the number and proportion of SIDS deaths in period 2 (2.4%) compared to those of period 1 (0.9%). This was followed by a decrease in numbers of SIDS in period 3, but an increase in the proportion of total deaths (3.4%) (Figures 2A,B). The increase in SIDS cases in the second period was the opposite of the decrease in SIDS cases in other countries in Europe. While the average SIDS per 1000 live births was 0.235, males were more likely to die of SIDS (0.268) than females (0.2), (Figure 3).

Among the explained deaths, the proportion in which infection was identified decreased significantly from 13.6% in period 1 to 8.4% in period 3 (*X*^2^ = 168.9, df = 2, *p* = 0). As noted in the methods, if severe inflammation or signs of septicemia were noted, these cases were not classified as SIDS. For SIDS, the proportion of infants with evidence of mild infection did not vary significantly: 66% in period 1; 65% in period 2, and 58% in period 3 (*X*^2^ = 4.57, df = 2, *p* = 0.1) (Table 3).

**Table 3:** **Analyses of evidence of infection in infant deaths in Hungary**.

	1979–1989 No (%)	1990–1999 No (%)	2000–2012 No (%)
Explained*	25873	13202	7584
Infection	3521 (13.6)	1451(10.9)	637 (8.4)
Unexplained (SIDS)	251	331	271
Mild infection	166 (66)	215 (65)	157 (58)

*Microbiologically confirmed and accepted.

A decrease in SIDS noted in the United Kingdom following initiation of infant immunization at 2 months rather than 3 months, particularly in the age range of 3–4 months. Between 1994 and 2012, there were 114 SIDS in the Budapest-Central Hungary area; 70 between 1994 and 2005 and 44 between 2006 and 2012. The immunization of infants at 2 months of age began in 2006. As with figures reported for the UK, the greatest decrease in SIDS was noted for children aged 3 months 14/70 (20%) prior to change in the immunization schedule to 2/42 (4.5%) in the second period (Fishers exact probability test, two tailed 0.026). While the numbers were small, they reflected the pattern noted in other studies.

## Discussion

In Hungary in the first and second periods examined, the main focus was to reduce infant mortality from known causes; it was much higher than in western European countries (1). The main strategy was to reduce perinatal mortality. Prevention of infections through immunization was an important part of this strategy and deaths from infection have declined (Table 1; Figure 2). These efforts were reflected in reduction in deaths due to meningitis, septicemia, and pneumonia. In contrast to other European countries, there was an increase in the proportion of SIDS in period 2 (1990–1999), followed by a decrease in period 3. For periods 1 and 2, evidence of mild infection was noted for over 60% of unexplained infant deaths investigated compared to 13.6 and 10.9% for infectious deaths in the comparable periods. In studies in the 1990s, *Streptococcus pneumoniae* was isolated from a significantly higher proportion of SIDS infants (11%) compared to healthy infants matched for age, sex, and locality (10). While rare, pneumococcal infections have been implicated in sudden infant deaths (11). It was noted in our data that there was a sharp decline in SIDS following introduction of the pneumococcal vaccine in 2008 (Figure 3) and it has remained below the numbers prior to 2008. Although the numbers were small, the decrease in the numbers of deaths at 3 months also decreased dramatically following initiation of infant immunization at 2 months in 2006 and, while the numbers are small they have not returned to levels noted prior to 2006.

There was no year when the SIDS rate per 1000 live births was more than 0.4/1000. The increase in SIDS in Hungary was not expected and requires investigation. There are several factors to be considered.

In the 1990s, there was a campaign based on studies in other countries to reduce the risk factors for SIDS. There have been no case-control studies in Hungary similar to those in other countries on which the “reduce the risks” campaigns were based. In Hungary, the prone sleeping position was not widely accepted as a real risk factor for SIDS. The risk factors for SIDS most widely accepted as significant were infection, lack of immunization, crowded living conditions associated with lower social-economic status, and exposure to cigarette smoke.

Reduction of exposure of infants to cigarette smoke was strongly emphasized in the campaign; however, compared to statistics available for Britain, the proportion of women in Hungary who smoke has not changed from the mid-1970s. Smoking among women decreased in the UK from 41% in 1974 to 17% in 2013 (12). For Hungary, there has not been a similar decline: 22% of women smoked in 1975 and 24% smoked in 2012 (13).

During and after the campaign, pathologists and forensic pathologists began to accept more widely that SIDS is a credible diagnosis. Documentation and the scene of death investigation became much more rigorous, not only to exclude infanticide or accidents but also to obtain data on symptoms of illness prior to death, prenatal and postnatal diseases, and disorders in development. Investigation focused not only on the pathomorphological changes but also evaluated the history of pregnancy and delivery. Postmortem microbiology was also introduced.

Although there was no standard autopsy protocol, the autopsy and histology in every case of sudden infant death is compulsory. Due to the increase in emphasis on accurate diagnosis, the possibility of change in diagnosis or bias cannot be ruled out; however, the age range affected, the excess of male deaths, and the pathomorphology (congested lungs, number and distribution of petechiae, liquid heart blood) were similar to those observed in Hungary prior to the intervention and in other countries. While the proportion of infection-related explained deaths declined during the three periods studied, the proportion of SIDS infants with evidence of minor infection/inflammation remained constant at approximately 60%.

There is evidence of infection in many of these infants. Bacteria or nuclear bodies in some indicating viral infections can be demonstrated microscopically. Bacterial and viral cultures have not been given much weight in assessment of SIDS because of the concern that the organisms are contaminants or overgrowth of normal flora and not related to the cause of death. More recent studies have identified bacteria in normally sterile sites in investigations of sudden death in infancy (14–16). Most of these organisms do not continue to grow when a body is stored at 4°C. In addition, staphylococcal toxins produced only at temperatures between 37 and 40°C have been identified in significant proportions of SIDS infants from different countries including Hungary (17) and toxigenic enteric organisms have also been identified in these infants (Bettelheim and Goldwater, this issue). Recent studies have indicated that virus infections might enhance inflammatory responses to mild bacterial infections (Moscovis et al., this issue). Autopsies need to include assessment of the molecular signs of inflammation such as cytokines that can affect the various physiological mechanisms proposed to explain these deaths (Blackwell et al., this issue).

In recent years, the autopsy rates in many countries have decreased, including Hungary. This is unfortunate as there is a growing persuasive argument for including new investigative techniques that could contribute to explaining the cause of these deaths: genetic investigations (Morris, this issue); and detailed microbiological investigations, both conventional diagnostic procedures and more extensive molecular screening techniques (Goldwater, this issue; Bettelheim and Goldwater, this issue).

## Conclusion

The current study compared explained deaths due to infection and those due to SIDS in three periods: 1979–1989 when the incidence of SIDS was higher in western Europe; 1990–1999 when the incidence of infant deaths was falling following introduction of the public health campaigns to reduce the risk factors identified for SIDS (e.g., prevention of prone sleeping, overheating, and exposure to cigarette smoke); and 2000–2012 to determine if introduction of Hib or pneumococcal vaccines had an effect on SIDS. There were reductions in SIDS cases identified following introduction of the Hib vaccine in 1999, introduction of the earlier immunization schedule in 2006, and introduction of immunization for pneumococcus in 2008. While reduction of prone sleeping or overheating cannot be ruled out as contributing to these decreases, the proportion of mothers who smoked remained constant. The evidence for infection/inflammation in explained deaths declined significantly over the three periods studied. Evidence of inflammation/infection in SIDS cases remained steady. Initiation of infant immunization in the United Kingdom at 2 months was associated with a shift in the age ranges of SIDS infants (7, 8), and although the numbers were small, there was a similar pattern of a sharp decline among infants who died at 3 months of age.

According to a recent study, the component of the postmortem examination that was the most helpful in diagnosis was the histological examination, followed by macroscopic examination, microbiological investigations, and clinical history. The majority of infection-related diagnoses were identified primarily by histological sampling rather than microbiological analyses, although microbiology aided in a diagnosis for 20% of cases that would have otherwise gone undetected (14). While autopsy findings have varied, signs of inflammation and responses to infection have appeared out of proportion to preexisting symptoms. The increase in inflammatory markers found in SIDS infants indicates that infection and inflammation might contribute to some of these deaths, either as a direct cause or the trigger of a lethal event. It might also be indicative of vulnerability in the immune response to an infectious trigger in infants. The protective effects of immunization (18, 19) noted for SIDS is further evidence for infection/inflammation playing a role in these infant deaths.

## Author Contributions

Each of the authors made substantial contributions to the conception, design, analyses, and interpretations of the work. They assisted in preparing the article, critically assessed the final version, and agree to be accountable for the accuracy and integrity of the work.

## Conflict of Interest Statement

The authors declare that the research was conducted in the absence of any commercial or financial relationships that could be construed as a potential conflict of interest.
